# Efficacy of donepezil for the treatment of oxaliplatin-induced peripheral neuropathy: DONEPEZOX, a protocol of a proof of concept, randomised, triple-blinded and multicentre trial

**DOI:** 10.1186/s12885-022-09806-8

**Published:** 2022-07-07

**Authors:** Nicolas Kerckhove, David Tougeron, Côme Lepage, Denis Pezet, Karine Le Malicot, Manon Pelkowski, Bruno Pereira, David Balayssac

**Affiliations:** 1 UMR 1107 NEURODOL, service de pharmacologie médicale, CHU Clermont-Ferrand, Université Clermont Auvergne, INSERM, 63000 Clermont-Ferrand, France; 2 Service d’Hépato gastroentérologie, CHU Poitiers, 86000 Poitiers, France; 3 Service d’Hépatogastroentérologie et oncologie digestive, CHU Dijon, Université de Bourgogne, Dijon, France; 4 UMR LNC 1231, EPICAD INSERM, Université de Bourgogne, Dijon, France; 5 Service de chirurgie digestive, U1071, M2iSH, USC-INRA 2018, CHU Clermont-Ferrand, Université Clermont Auvergne, INSERM, INRA, 63000 Clermont-Ferrand, France; 6 Fédération Francophone de Cancérologie Digestive (FFCD), 21079 Dijon, France; 7 Direction de la recherche clinique et de l’innovation, CHU Clermont-Ferrand, 63000 Clermont-Ferrand, France

**Keywords:** Pain, Chemotherapy-induced peripheral neuropathy, Anticholinesterase, Donepezil, Oxaliplatin, Study protocol

## Abstract

**Background:**

The use of oxaliplatin in digestive tract cancers could induce severe peripheral neuropathy (OIPN) decreasing the quality of life of patients and survivors. There is currently, no univocal treatment for these peripheral neuropathies. Donepezil, a reversible inhibitor of cholinesterase, used to treat Alzheimer’s disease and dementia, is reported to have a good safety profile in humans, and preclinical data have provided initial evidence of its effectiveness in diminishing neuropathic symptoms and related comorbidities in OIPN animal models.

**Methods:**

The DONEPEZOX trial will be a proof-of-concept, randomised, triple-blinded, and multicentre study. It will be the first clinical trial evaluating the efficacy and safety of donepezil for the management of OIPN. Adult cancer survivors with OIPN that report sensory neuropathy according to QLQ-CIPN20 sensory score (equivalence of a grade ≥ 2), at least 6 months after the end of an oxaliplatin-based chemotherapy will be included. Eighty patients will be randomly assigned to receive either donepezil or placebo over 16 weeks of treatment. The primary endpoint will be the rate of responders (neuropathic grade decreases according to the QLQ-CIPN20 sensory score) in the donepezil arm. The severity of OIPN will be assessed by the QLQ-CIPN20 sensory scale before and after 16 weeks of treatment. The comparison versus the placebo arm will be a secondary objective. The other secondary endpoints will be tolerance to donepezil, the severity and features of OIPN in each arm before and after treatment, related-comorbidities and quality of life. Fleming’s one-stage design will be used for sample size estimation. This design yields a type I error rate of 0.0417 and power of 91% for a responder rate of at least 30% in donepezil arm. A total of 80 randomized patients is planned.

**Discussion:**

This study will allow, in the case of positive results, to initiate a phase 3 randomized and placebo-controlled (primary endpoint) clinical study to assess the therapeutic interest of donepezil to treat OIPN.

**Trial registration:**

NCT05254639, clincialtrials.gov, Registered 24 February 2022.

## Background

Chemotherapy-induced peripheral neuropathy (CIPN) is a common adverse effect of neurotoxic anticancer drugs (platinum salts, taxanes, vinca alkaloids, bortezomib, thalidomide), which impacts the patients’ quality of life [1]. CIPN occurs in approximately 68% of patients treated with multiple agents [2]. The incidence depends on the chemotherapy regimen, the duration of exposure, and assessment methods [1, 3]. CIPN is the consequence of damage to the peripheral nervous system caused by neurotoxic anticancer agents. These CIPN are mainly characterized by symmetrical sensory disorders with a distal predominance and without truncal or radicular systematization. Most of the symptoms of CIPN correspond to a clinical picture of sensory polyneuropathy, causing loss of sensitivity, paresthesia, dysesthesia, burning sensations, altered fine motor skills and neuropathic pain. About 30% of patients with sensory CIPN also suffer from neuropathic pain [4, 5]. Motor symptoms such as cramping, twitching, muscle atrophy and vegetative disorders can also occur [1]. With cumulative doses, symptoms progress from the ends of the hands and feet to the wrists and ankles and finally the arms and legs [6]. In addition, the perioral or face region, and auditory system can also be affected [7–9].

Oxaliplatin is widely used to treat digestive tract cancers despite being extremely neurotoxic. This neurotoxicity is often dose-limiting and hampers the efficacy of chemotherapy [10]. Oxaliplatin-induced peripheral neuropathy (OIPN) may occur either shortly after oxaliplatin infusion (up to 90% of patients) [2] characterized mainly by acute and transient cold hyperalgesia, or as late-onset chronic cumulative peripheral neuropathy (30–50% of patients) [11]. The resulting neuropathy symptoms can be persistent and definitive (> 24 months [11] and > 48 months [12, 13]), and associated with psychological distress (anxiety and depression) and a decrease of patients’ health-related quality of life (HRQoL) [14].

Although a number of compounds have been investigated to prevent or treat CIPN, none of them are sufficiently effective to be used routinely in clinical practice [15, 16]. Only duloxetine was recommended by ASCO and ESMO for neuropathic pain-related to CIPN [15, 16]. As a result of the lack of clear evidence, the current strategies used to reduce CIPN essentially consist in modifying of the chemotherapy doses and infusion schemes, although sometimes it continues to worsen despite stopping oxaliplatin. This aggravation can be linked to risk factors such as diabetes and excessive alcohol consumption. Little or no follow-up or treatment is offered to patients developing chronic neuropathy after their chemotherapy [10, 13, 17]. This leads to medical wandering, which is very harmful for the patient. Lastly, OIPN is one reason why, despite negative results of the IDEA trial [18], oxaliplatin-based adjuvant treatment of high-risk stage II and low-risk stage III colon cancers is now used for only 3 months as standard of care as compared the previously 6 months of adjuvant treatment.

Donepezil, a reversible inhibitor of cholinesterase, is used to treat Alzheimer’s disease and dementia [19]. Donepezil is reported to have a good safety profile in humans [20–22]. In addition, preclinical data have provided initial evidence of its effectiveness in diminishing neuropathic symptoms and related comorbidities in OIPN animal models [23, 24]. The preclinical evidence and the lack of clinical trial data led us to design the first proof of concept study to assess the efficacy and tolerance of donepezil in the treatment of OIPN in cancer survivors.

### Preclinical & clinical arguments for using donepezil to treat OIPN

Preclinical works have demonstrated that donepezil was able to prevent and treat tactile allodynia in oxaliplatin-treated animals [23, 24]. More precisely, OIPN was responsible of an increase of the cholinergic neurotransmission involving acetylcholine muscarinic M2 receptors (m2AChRs) in the insular cortex of oxaliplatin-treated rats. In the insular cortex, choline (acetylcholine metabolite) concentration was significantly increased and correlated to mechanical allodynia. Moreover, m2AChRs were also up-regulated, and insular micro-injection of oxotremorine (m2AChRs agonist) suppressed mechanical allodynia. Finally, the antineuropathic effect of donepezil was blocked by insular micro-injection of methoctramine (m2AChRs antagonist) [24]. Interestingly, data have highlighted the beneficial effect of donepezil on neuropathic symptoms in different neuropathic pain conditions, likely found in patients affected by CIPN, without significant adverse effects. Likewise, several studies demonstrated that donepezil induces analgesic and neuroprotective effects [25–29]. Recently, a preclinical study demonstrated that donepezil induced an antineuropathic effect in diabetic mice with neuropathic pain [30].

Clinical studies have confirmed the potential antineuropathic effect of donepezil. Boyle et al*.* [31] showed in healthy volunteers that donepezil (associated with gabapentin) reduced the pain thresholds (better than gabapentin alone) caused by the stimulation of the sural nerve without severe adverse effects. Similarly, this result was also observed in two studies with patients suffering from various neuropathic pain [32, 33]. Finally, a case report demonstrated an analgesic effect of donepezil in a patient with painful Alzheimer’s disease [34]. Moreover, donepezil improved depression and anxiety scores and HRQoL in cancer patients treated by opioids [35].

All of these clinical and preclinical data have thus highlighted the potential beneficial effect of donepezil on neuropathic symptoms, without any severe adverse effect. We therefore hypothesized that the use of donepezil could reduce the symptoms of OIPN and limit the decrease in HRQoL and reduce psychological distress (anxiety and depression) in digestive cancer survivors.

### Mechanism of CIPN

Better knowledge of the neurotoxicity mechanism induced by anticancer agents is now available. Peripheral neuropathy can result from various causes: damage of the microtubule transport of axons; distal axonal degeneration [36]; morphological change of the nuclei of dorsal root ganglia (DRG) neurons; alteration of mitochondrial function in axons; and intra-axonal accumulation of sodium and calcium by disruption of voltage-gated sodium (Nav) and potassium channels [37]. Nerve damage depends on the anticancer drug administered. Oxaliplatin is known to damage the nuclei of DRG neurons; cause channelopathies of the Nav channel (variation of expression and functionality) and cold receptors of the transient receptor potential family (TRPM8 and TRPA1) [38]. An increase in the glutamate level in the spinal dorsal horn has also been observed in oxaliplatin-treated animals [39], suggesting an involvement of glutamatergic neurotransmission in pain hypersensitivity development induced by oxaliplatin. The chronic sensory form is considered to be induced by morphological and functional changes in the DRG, resulting from the local deposition and accumulation of oxaliplatin. Mitochondrial toxicity could represent an important pathophysiological basis for the chronic neurotoxicity of platinum derivatives [40], as well as satellite glial cell activation in DRG [41].

### Current treatment available for CIPN

Several therapeutic agents (antioxidants, neuroprotective agents, analgesics, anticonvulsants, antidepressants, dietary supplements, etc.) have been found promising for CIPN treatment [42]. However, the evidence of their effectiveness remains controversial and the treatment of CIPN is still largely symptomatic. Antidepressants, antiepileptics and current analgesics are the only options available to clinicians, mainly for treating neuropathic pain symptom; however, their effectiveness is limited and associated with significant adverse events like drowsiness. Two studies demonstrated the potential interest of duloxetine for managing CIPN [43, 44], but these studies assessed only the neuropathic pain component (which represents about 30% of patients with CIPN [4, 5]), or they were not randomized or controlled. Lastly, a recent study has shown the preventive effect of Renin-Angiotensin System Inhibitors but this must be confirmed in a randomized controlled trial [45]. The recent update of ASCO and ESMO guidelines for the prevention and treatment of CIPN states that no treatment can be recommended for the prevention of CIPN and only duloxetine can be recommended (level: moderate) for the treatment of neuropathic pain related to CIPN [15, 16]. Therefore, to avoid severe CIPN, dose reduction or discontinuation of the neurotoxic anticancer drug is the only option to limit the progression of CIPN [46, 47].

Donepezil, marketed for the treatment of Alzheimer’s disease [48], is known to be neuroprotective [49, 50], anxiolytic and antidepressant [51], with a known safety profile based on Alzheimer patients, and may thus be a suitable candidate for treatment of CIPN.

## Methods / design

### Study design

The present study is a randomised, triple-blinded (patients, clinicians and pharmacists), and multicentre proof-of-concept trial that assesses the efficacy and safety of donepezil in patients with OIPN. The plan is to include 80 patients from 26 cancer centres and hospitals in France. The duration of the study is 20 weeks for the patients, including 4 weeks for treatment initiation, 12 weeks of stable treatment and 4 weeks of follow-up.

### Study objectives

The primary objective is to assess the curative efficacy of donepezil on the severity of OIPN in patients having received and completed oxaliplatin-based chemotherapy indicated for colorectal and pancreatic cancers and eliciting QLQ-CIPN20 sensory score ≥ 30 / 100 (equivalent to grade ≥ 2 peripheral neuropathy). Efficacy will be based on the percentage of responders (decrease of peripheral neuropathic grade according to the QLQ-CIPN20 sensory score) only in the donepezil arm (see the “Study endpoints” chapter for details).

The secondary objectives are safety (type, intensity, frequency of adverse effects and treatment discontinuation rate due to adverse effects) of donepezil, and its efficacy compared to the placebo arm, for:

- Neuropathic symptoms throughout the study (severity and responders),
- Anxiety and depression,
- OIPN grade,
- Neuropathic pain,
- Health-related quality of life,
- Patient global impression of change (PGIC),
- Consumption of painkillers.

### Inclusion and exclusion criteria

Participants will be adult cancer survivors with OIPN diagnosed for more than 3 months and not relieved by the usual treatments.

#### Inclusion criteria

- ◦ Adult Male / Female who received oxaliplatine-based chemotherapy for colorectal or pancreatic cancers,
- ◦ QLQ-CIPN20 sensory score ≥ 30 / 100 (equivalent to the National Cancer Institute—Common terminology criteria for adverse events (NCI-CTCAE) v4.0 grade of neuropathy ≥ 2),
- ◦ Diagnosis of OIPN treated or not by stable antineuropathic treatment (opioids, pregabalin, gabapentin, duloxetine and other antidepressants or anticonvulsants) for at least 1 month,
- ◦ Chemotherapy completed for at least 6 months,
- ◦ Patients affiliated with the French national health insurance,
- ◦ Written informed consent,
- ◦ French language comprehension.

#### Exclusion criteria

- ◦ Ongoing cancer,
- ◦ Pregnancy or breastfeeding (required contraception),
- ◦ Patient with chronic progressive disease inducing pain (excluding OIPN),
- ◦ Diabetic patient (excluding non-insulin-treated or insulin-treated diabetes of less than 5 years) or presence of a proven diabetic neuropathy
- ◦ Other types of neuropathies,
- ◦ ALT / AST more than 3 times the normal value,
- ◦ Severe cardiovascular disease, bradycardia (< 55 bpm), heart conduction disorders,
- ◦ History or active gastroduodenal ulcer,
- ◦ Asthma or obstructive pulmonary disease,
- ◦ Allergy to donepezil or piperidine derivatives,
- ◦ Drug interactions: CYP3A4 inhibitors (ketoconazole, itraconazole and erythromycin); CYP2D6 inhibitors (quinidine, fluoxetine) and enzymatic inducers (rifampicin, phenytoin, carbamazepine),
- ◦ Dependence on alcohol and/or drugs,
- ◦ Psychotic disorders, patient under antipsychotics.

No therapeutic change will be generated by the protocol; patients will be treated with donepezil or placebo in addition to their current treatment of OIPN, if any. However, no therapeutic change for OIPN or pain will be allowed throughout the study.

Patients can discontinue the study treatment for any of the following reasons: intolerance to donepezil and significant adverse events; and can be withdrawn from analysis for any of the following reasons: modification of the antineuropathic / analgesic therapy (if any) during the study, withdrawal of consent and serious protocol deviation.

### Investigational Medicinal Product (IMP)

#### Donepezil Mylan®

The study dose of donepezil (5 mg tablet, MYLAN) will be used according to the recommendations by summary of product characteristics (SPC) and will be identical to the dose approved for treating Alzheimer disease and dementia. According to the SPC of donepezil, the dose of 10 mg/day (2 tablets of 5 mg as a single dose) will be reached after 4 weeks at the dose of 5 mg/day. However, if the patient presents efficacy (decrease of OIPN grade) or adverse effects that appear to be related to the study treatment, the investigator will have the option of continuing treatment at a dose of 5 mg/day, until the end of the study.

To improve adherence to intervention protocols, drug tablet return will be counted by pharmacists and during monitoring.

#### Placebo

The donepezil and the placebo tablets will be in identical opaque blister and will have the same shape, the same colour and the same branding. The placebo will be taken for 16 weeks with the same administration modalities as the donepezil group.

Patients will be given a diary to remind them of their treatment dosage and to indicate any deviations in their intake. Adverse events will be collected by patients throughout the study in their diaries and during the various visits. The type, intensity and potential relationship with the study treatment will be informed.

Treatment should be discontinued if:

- AST or ALT levels ≥ 3 times normal values,
- Appearance of severe cardiovascular disease (as determined by clinician), bradycardia (< 55 bpm), cardiac conduction disturbances (assessed by electrocardiogram),
- Development of peptic ulcer disease,
- Development of asthma or obstructive lung disease,
- Development of an allergy to donepezil or piperidine derivatives.
- Pregnancy

Investigators will be allowed to unblind without sponsor intervention in case of emergency (e.g. serious adverse event, death, pregnancy).

### Study endpoints

#### Primary endpoint—QLQ-CIPN20 questionnaire (sensory scale)

The EORTC was developed the QLQ-CIPN20 questionnaire as an auto-questionnaire specifically to assess CIPN severity. The QLQ-CIPN20 contains 20 questions about the patients’ experience regarding functional limitations and symptoms related to their CIPN. This auto-questionnaire consist of 3 subscales: sensory, motor and autonomic, which permit a comprehensive picture of the characteristics of CIPN. This auto-questionnaire has been previously validated in clinical trials [52, 53]. The literature suggests that the assessment of CIPN symptoms is preferable with Patient-Reported Outcomes (PROs) rather than Clinician-Reported Outcomes (CROs). Moreover, the QLQ-CIPN20 questionnaire is considered as one of the most appropriate outcome measures [54–56].

The sensory subscale assesses various symptoms such as paraesthesia, dysesthesia, numbness and pain; and is more relevant to assess CIPN than the 11-point numerical rating scale (NRS) for pain, which is specific only for pain symptoms which affects only 30% of patients [4, 5]. Other studies have demonstrated that the comparison of QLQ-CIPN20 and the NCI-CTCAE sensory neuropathic grade provides convergent validity [57, 58]. The QLQ-CIPN20 also provided more detailed information, permitting to distinguish more severity degrees of neuropathy, and was more responsive to change over time than the NCI-CTCAE [58]. Finally, as our study will be a multicentre trial, using the QLQ-CIPN20 will make it possible to carefully standardize the assessment of OIPN between centres.

For the primary endpoint (percentage of responders to the treatment), only the sensory scale scores will be used. According to the studies of Alberti et al*.* [57] and Le-Rademacher et al*.* [58], the sensory scale score can be correlated with the NCI-CTCAE sensory neuropathy grade (QLQ-CIPN20 scores < 30: grade 1, ≥ 30 and ≤ 40: grade 2, and > 40 / 100: grade 3–4).

A responder will correspond to a patient moving from a score ≥ 30 and ≤ 40 to a score < 30 / 100 or from a score > 40 to a score ≤ 40 / 100.

Because of the absence of preliminary clinical results in the literature about donepezil and CIPN, and the recommendations of international experts (expertise of our project in the framework of a call for projects), we will perform a proof-of concept study to generate preliminary results for a future randomised placebo-controlled phase 3 study if the results are positive. Then, for the primary endpoint, the responder rate will not be compared to placebo, but assessed only for the donepezil arm after 16 weeks of treatment. Donepezil will be considered effective if 7 or more responders are observed in the 33 patients of the donepezil arm (see “statistics” paragraph for details). The responder rate will be compared to the placebo arm only for the secondary endpoint.

In the event of a beneficial therapeutic effect at the end of treatment (significant reduction in neuropathic disorders [reduction of one grade of neuropathy (according to NCI-CTCAE v4.0 or QLQ-CIPN20 score) or -30% of neuropathic pain (according to NRS 0–10 scale), if present, or PGIC score of 6 or 7]), the investigator will be allowed to request a lifting of the treatment blind, to adapt the further management of the patient and/or refer the patient to a specialist (neurologist, pain physician).

#### Secondary endpoints

The evolution of all the secondary parameters will be studied in an intragroup (before *vs.* after treatment) and an intergroup (placebo *vs.* donepezil arm): i) Sensory, motor and autonomic scales from the QLQ-CIPN20 questionnaire (see below); ii) Grading of OIPN by NCI-CTCAE v4.0: this scale will be used to grade the severity of sensory neuropathy from 1 to 4 (1 = Asymptomatic, 2 = Moderate symptoms; limiting instrumental of Activities of Daily Living [ADL], 3 = Severe symptoms; limiting self-care ADL, 4 = Life-threatening consequences; urgent intervention indicated); iii) Assessment of neuropathic pain: monthly assessment of pain felt in the last week by 11-point NRS. This scale is used for adults and children 10 years old or older patient self-reporting of pain (score of 0 = no pain and 10 = worst possible pain). If NRS ≥ 4/10, neuropathic pain will be validated by the DN4 interview questionnaire (“*Douleur Neuropathique 4*”) will be mandatory. The interview portion of the DN4 questionnaire is a clinician-administered screening tool for neuropathic pain [59]. The questionnaire includes 7 items (YES or NO, the test is positive for a score ≥ 3/7). If the patient have a positive DN4 and a pain intensity ≥ 4/10 (NRS), neuropathic pain will be confirmed and will be characterized by the Neuropathic Pain Symptom Inventory (NPSI) questionnaire. This self-reported questionnaire assesses neuropathic pain symptoms [60], and includes 12 items; iv) HRQoL by the QLQ-C30 questionnaire (EORTC): this auto-questionnaire (EORTC) assesses the HRQoL of cancer patients, and includes five functional scales, three symptom scales, an overall HRQoL scale, and a number of additional elements assessing other symptoms; v) Anxiety and depression by the hospital anxiety and depression scale (HADS) questionnaire: this auto-questionnaire detects anxiety and depressive disorders, and includes 14 items rated from 0 to 3 (7 questions for anxiety and 7 for depression). The following interpretation is proposed for each of the two total scores: ≤ 7: absence of symptomatology; 8 to 10: suggestive symptomatology; ≥ 11: indicative symptomatology; vi) Patients’ Global Impression of Change (PGIC): PGIC [61] assesses the general effectiveness of the treatment according to the impression of patient, with the scores from 1 to 7 (from aggravated to very improve); vii) Safety: the time to treatment failure will be assess. This is the interval between randomization to treatment discontinuation for any reason. The assessment of adverse events (AEs) associated with study treatment will be also assessed by NCI-CTCAE v4.0. Any AEs will be collected daily by the patient and during the monthly visits. AEs will be categorized according to their intensity, treatment-related or not and by type. At last, viii) Assessment of painkiller consumption (if any): drug consumption for OIPN will be assessed throughout the study.

### Methodology and study design (Fig. 1)

**Fig. 1:**
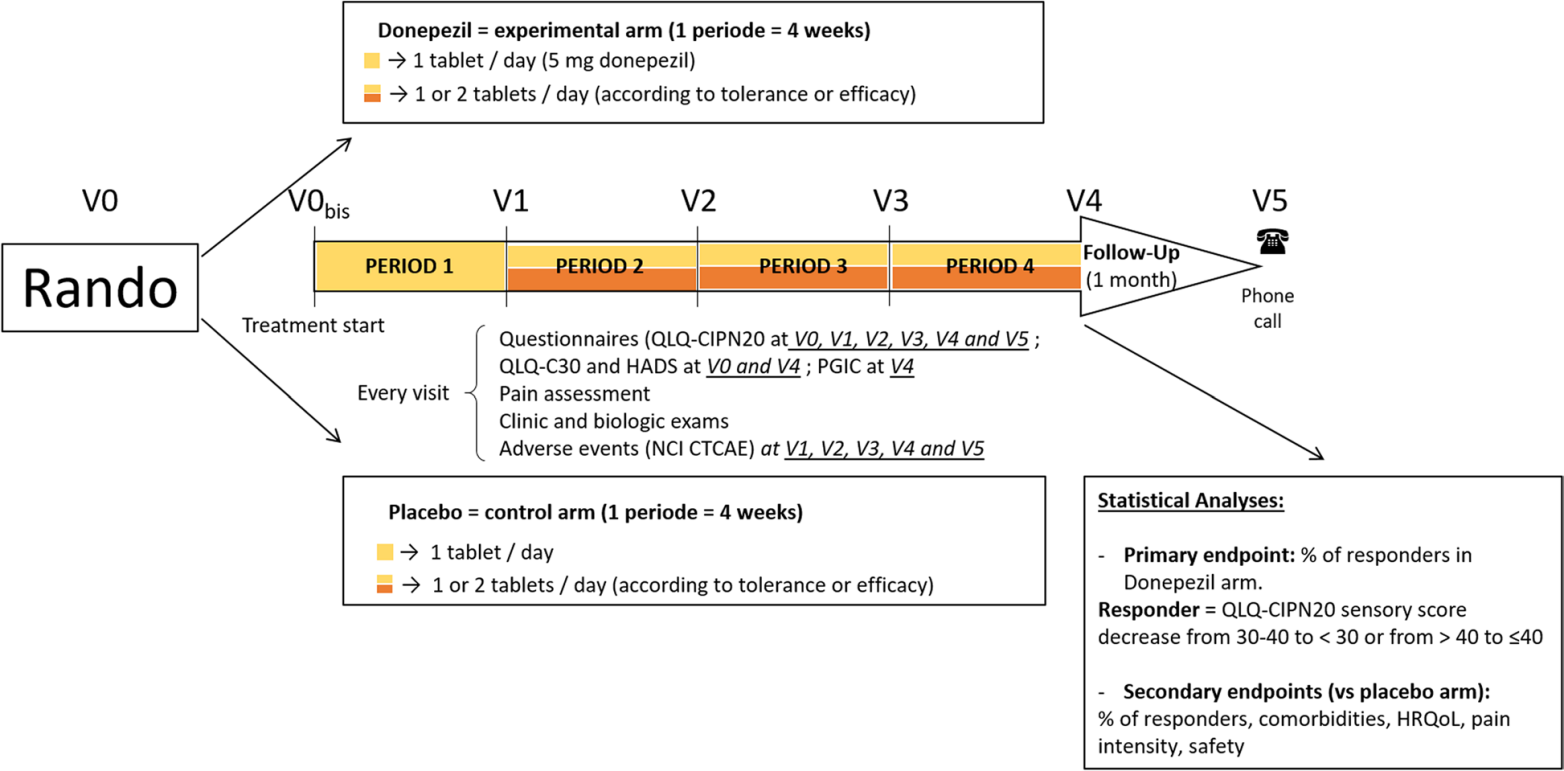
Study Design and evaluations

#### Enrolment

Colorectal and pancreatic cancer survivors having received oxaliplatin-based chemotherapy and eliciting peripheral neuropathy of a grade ≥ 2 (QLQ-CIPN20 sensory scores ≥ 30 / 100) will be included by clinicians to receive either placebo or donepezil. Patients will either be contacted by phone to propose the study or during one of their consultations at the clinical centre.

#### Inclusion/randomization visit (V0)

The study will be presented to the patient, along with the objectives, organization, constraints, and assessment questionnaires. If the patient accepts to participate, they will confirm it by signing the informed consent form prior to any study procedure. The following will be performed:

- Validation of patient eligibility (inclusion and non-inclusion criteria),
- Clinical examination and body weight,
- Electrocardiogram and heart rate,
- Blood sampling (hepatic and renal function, complete blood count),
- Verification of presence of OPIN (QLQ-CIPN20),
- Randomization,
- Completion of study questionnaires (HADS and QLQ-C30),
- Neuropathic pain assessment: 11-point NRS, and if pain score ≥ 4 / 10 then perform DN4 interview and complete NPSI questionnaire if DN4 interview positive (score ≥ 3 / 7),
- Assessment of painkiller consumption (if applicable),
- Delivery of study treatment according to randomization for a 4-week period: donepezil or placebo.

#### Visits after 4, 8 and 12 weeks of initiation treatment (V1, V2 and V3)

The following will be performed:

- The patient will return the empty or unused study treatment from the last treatment period,
- Completion of QLQ-CIPN20 questionnaire and 11-point NRS pain,
- Assessment of painkiller consumption (if applicable),
- Electrocardiogram and heart rate,
- Blood sampling (hepatic function, complete blood count),
- Body weight,
- Assessment of safety (adverse effects by NCI CTCAE v4.0),
- Determination of the dose to be delivered according to the treatment efficacy and tolerance, for the 4-weeks period. In the case of efficacy or poor tolerance, the dose will remain at 5 mg/day. If the study treatment is well tolerated but does not show efficacy (according to QLQ-CIPN20 score), the dosage will be increased to 10 mg/day.

#### End of study treatment visit (V4)

The visit will take place after 16 weeks of treatment or after study treatment discontinuation. The following will be performed:

- The patient will return the empty or unused study treatment from the last treatment period,
- Clinical examination to assess the grade of neuropathy (NCI-CTCAE v4.0),
- Completion of study questionnaires (QLQ-CIPN20, HADS and QLQ-C30),
- Neuropathic pain assessment: 11-point NRS, and if pain score ≥ 4 / 10 then perform the DN4 interview and complete the NPSI questionnaire if the DN4 interview is positive (score ≥ 3 / 7),
- Assessment of painkiller consumption (if applicable),
- Electrocardiogram and heart rate,
- Blood sampling (hepatic function, complete blood count),
- Body weight,
- Assessment of safety (adverse effects by NCI CTCAE v4.0).

#### Post study treatment follow-up phone call

This phone call will be planned 1-month after the “End of study treatment visit”.

The following will be performed during the post-treatment phone call:

- Neuropathic pain assessment: 11-point NRS, and if the pain score ≥ 4 / 10 then the perform DN4 interview and complete the NPSI questionnaire if the DN4 interview positive (score ≥ 3 / 7),
- Completion of study questionnaires (QLQ-CIPN20),
- Assessment of painkiller consumption (if applicable),
- Evaluation of safety (adverse effects by NCI CTCAE v4.0).
- All the questionnaires will be given to the patients during V4 to allow them to complete the questionnaires at home after 1-month. The phone call will remind the patients to return the completed questionnaires to the investigating centre.

### Statistical considerations

#### Sample size estimation

Fleming’s one-stage design will be used for sample size estimation. Sixty-six evaluable patients (33 by arm) will be accrued. The null hypothesis that the responder rate is 10% (maximal non-efficacy threshold, according to Smith et al*.* [43]) will be assessed against a one-sided alternative for the donepezil arm. The null hypothesis will be rejected if 7 assessable or more responders are observed in the 33 patients of the donepezil arm. This design yields a type I error rate of 0.0417 and a power of 91% for a responder rate of at least 30% in the donepezil arm (minimal clinical efficacy threshold). Thus, we have decided to include a total of 80 patients (2 × 40) to take into account lost to follow-up (20%, according to clinical practice with Alzheimer patients, which mainly corresponds to a poor tolerance at 5 mg/day dosage or when switching from 5 mg/day to 10 mg/day dosage), and possible tumor recurrence.

#### Statistical analysis

All statistical analyses will be performed using SAS software version 9.4 before breaking the randomization codes, according to International Conference on Harmonization-Good Clinical Practice guidelines. A two-sided *p*-value of less than 0.05 will be considered for the statistical significance of all the analyses. The results will be expressed as hazard-ratios and 95% confidence intervals. Secondary analyses will be exploratory.

The baseline description of the patients included in the analysis will be carried out, without presenting inferential statistical tests (according to recommendations). As part of a proof-of-concept, only patients who have completed the study (treatment received in its entirety for 16 weeks whatever the dose = per protocol population) will be analyzed for the primary endpoint. The primary endpoint will be the rate of responders and will be described using frequency and percentage. A 95% one-sided confidence interval will also be calculated. Subgroup analyses for the primary endpoint will be planned for each stratum of the randomization (neuropathy grade, age of neuropathy and type of chemotherapy).

For the secondary endpoints, analyses will be performed on the intention-to-treat and per-protocol populations and compared to the placebo arm.

Descriptive statistics will be presented by treatment arms. Continuous variables will be presented as mean and standard deviation (otherwise as median and interquartile range) and will be compared between arms using the unpaired t test or the Mann–Whitney U test when appropriate according to the assumptions of the t-test: (i) the Shapiro–Wilk test will be used to assess normality, and (ii) the Fisher-Snedecor test to assess homoscedasticity. When appropriate (for example, for the sensory scores of the QLQ-CIPN20), these analyses will be supplemented by the recommendations proposed by Vickers and Altman, i.e. ANCOVA, considering baseline scores as covariates. Then, multiple linear mixed models could be run with centers as random-effects. Covariates will be determined according to univariate results and to their clinical relevance, including stratification parameters. The normality of residuals will be studied as described previously. If appropriate, a transformation (for example logarithmic) should be proposed to achieve the normality of dependent variable. The results will be expressed as regression coefficients and 95% confidence intervals. The analyses of other continuous secondary endpoints (11-point pain NRS, DN4 interview, NPSI, HADS and QLQ-C30 questionnaires) will be carried out similarly. For categorical parameters (adverse effects, PGIC, DN4 interview or HADS categorized according to literature), Chi-squared or Fischer exact tests will be used to compare the treatment arms. A generalized linear mixed model (more precisely logistic regression for dichotomous outcomes) will be applied for multivariable analyses. The results will be expressed as relative risks and 95% confidence intervals.

Repeated data collected longitudinally will be analyzed by mixed models (linear or generalized linear according to dependent variable) to study the following fixed effects, arm, time and their interaction taking into account between and within patient variability (patient as random-effect, in addition to center effect) [62]. Particular attention will be paid to safety analysis (by group: type, intensity, frequency of adverse effects). Exit rates due to adverse effects will be considered as a censored data. These criteria will be estimated by the Kaplan–Meier method, compared between groups by the log-rank test, and investigated in a multivariate marginal Cox model considering center effects. The proportional-hazard hypothesis will be verified using Schoenfeld’s test and plotting residuals. Finally, to put significant results in perspective, a sensitivity analysis will be conducted to measure the impact of the missing data. A worst-case scenario will be defined as one where patients with missing data do not react to the treatment (whatever the treatment may be). Additional analyses will then be performed according to the statistical nature of missing data (missing at random or not), in particular multiple imputation or estimations proposed by Verbeke and Molenberghs [63] adapted specifically to repeated data.

## Discussion

This study did not encounter any major difficulties in its design and preparation. Nevertheless, there are important points to consider in the design of this type of trial in order to provide a result with an acceptable level of evidence for the scientific and medical community.

Among these points, we can mention the importance of having a comparison group and at least a double-blind study. Even if in our study, which remains a proof of concept, the comparison with the comparator arm remains a secondary objective, the choice of a placebo comparator group was motivated by three reasons. The first is that there is no active comparator. Indeed, to date, there is no gold standard for the treatment of OIPN [15, 64]. The second reason is the international recommendations of the European Medicines Agency (EMA) for the evaluation of analgesic treatment [65]. These recommendations indicate the need to use a placebo group. The last reason is the placebo effect, which remains very important for pain relief therapies [66]. Thus, the choice to keep a placebo group seemed important to us, and the results of the comparison between the two study arms, even if secondary, will bring a greater level of evidence to our results.

We can also mention the duration of the study. In accordance with the EMA and IMMPACT recommendations, a duration of at least 12 weeks of treatment is recommended to increase assay sensitivity and provide the basis for designing confirmatory trials [67].

It is also important to note the choice of the evaluation criteria. In this study, we chose the sensory score of the QLQ-CIPN20. As previously mentioned, this questionnaire is recommended in several studies for the evaluation of CIPN. Most importantly, this choice also standardizes the assessment of OIPN, thus avoiding measurement bias between centres.

Finally, the study should be as representative as possible of the target population and national clinical practices. For this purpose, 26 investigating centres located throughout France and including academic (regional and university hospitals) and private (cancer centre and private clinic) institutions were selected. It should be noted that a pre-selection of the centres was carried out in order to evaluate their capacity to carry out the study under the conditions of the protocol. The purpose of this selection was to increase the feasibility and success of our study.

Our study also has some limitations. The two main ones being the design in proof of concept and the comparison with the placebo arm only in secondary endpoint. Because of these limitations, the results of this study will have to be confirmed by a randomized, controlled, double-blind study comparing the donepezil arm with the placebo arm as primary endpoint.

Another limitation is the non-inclusion of patients with ongoing cancer. This choice was made because we do not yet have preclinical results on a possible drug interaction between donepezil and the antitumor efficacy of oxaliplatin. Thus, for patient safety, only patients who have completed their chemotherapy will be included. Finally, no electrophysiological or Quantitative Sensory Test exploration is planned in the study. This choice is purely logistical because the vast majority of investigating centres do not have the tools and personnel available to perform this type of exploration.

## Data Availability

The data set will be the property of the University Hospital of Clermont-Ferrand and FFCD. The principal investigator (DP) and the data-manager will have full access to the final data set. The results will be communicated in a peer-reviewed journal, presented at international congresses and completed online on ClinicalTrials.gov. The datasets used and/or analysed during the current study are available from the corresponding author on reasonable request.

## Abbreviations

AChRs: Acetylcholine Receptors
ADL: Activities of Daily Living
ALT / AST: Alanine aminotransferase / aspartate transaminase
ASCO: American Society of Clinical Oncology
CIPN: Chemotherapy-induced peripheral neuropathy
CROs: Clinician-Reported Outcomes
DN4: “*Douleur Neuropathique 4*”
DRG: Dorsal root ganglia
EMA: European Medicines Agency
EORTC: European Organisation for Research and Treatment of Cancer
ESMO: European Society for Medical Oncology
HADS: Hospital anxiety and depression scale
HRQoL: Health-related quality of life
IDEA: International duration evaluation of adjuvant therapy
IMMPACT: Initiative on Methods, Measurement, and Pain Assessment in Clinical Trials
Nav: Voltage-gated sodium
NCI-CTCAE: National Cancer Institute—Common terminology criteria for adverse events
NPSI: Neuropathic Pain Symptom Inventory
NRS: Numeric Rating Scale
OIPN: Oxaliplatin-induced peripheral neuropathy
PROs: Patient-Reported Outcomes
PGIC: Patient global impression of change
QLQ-CIPN20: Quality of life questionnaire—Chemotherapy-induced peripheral neuropathy 20
QLQ-C30: Quality of life questionnaire – Cancer 30
SPC: Summary of product characteristics
TRPA1: Transient receptor potential A1
TRPM8: Transient receptor potential M8
